# Targeted inhibition of the ATR/CHK1 pathway overcomes resistance to olaparib and dysregulates DNA damage response protein expression in *BRCA2*^MUT^ ovarian cancer cells

**DOI:** 10.1038/s41598-023-50151-y

**Published:** 2023-12-19

**Authors:** Łukasz Biegała, Arkadiusz Gajek, Izabela Szymczak-Pajor, Agnieszka Marczak, Agnieszka Śliwińska, Aneta Rogalska

**Affiliations:** 1 Department of Medical Biophysics, Institute of Biophysics, Faculty of Biology and Environmental Protection, University of Lodz, 141/143 Pomorska Street, 90-236 Lodz, Poland; 2 Doctoral School of Exact and Natural Sciences, University of Lodz, 21/23 Jana Matejki Street, 90-237 Lodz, Poland; 3 Department of Nucleic Acid Biochemistry, Medical University of Lodz, 251 Pomorska Street, 92-213 Lodz, Poland

**Keywords:** Ovarian cancer, DNA damage response, Cancer therapeutic resistance, Apoptosis

## Abstract

Olaparib is a PARP inhibitor (PARPi) approved for targeted treatment of ovarian cancer (OC). However, its efficacy is impeded by the inevitable occurrence of resistance. Here, we investigated whether the cytotoxic activity of olaparib could be synergistically enhanced in olaparib-resistant OC cells with *BRCA2* reversion mutation by the addition of inhibitors of the ATR/CHK1 pathway. Moreover, we provide insights into alterations in the DNA damage response (DDR) pathway induced by combination treatments. Antitumor activity of olaparib alone or combined with an ATR inhibitor (ATRi, ceralasertib) or CHK1 inhibitor (CHK1i, MK-8776) was evaluated in OC cell lines sensitive (PEO1, PEO4) and resistant (PEO1-OR) to olaparib. Antibody microarrays were used to explore changes in expression of 27 DDR-related proteins. Olaparib in combination with ATR/CHK1 inhibitors synergistically induced a decrease in viability and clonogenic survival and an increase in apoptosis mediated by caspase-3/7 in all OC cells. Combination treatments induced cumulative alterations in expression of DDR-related proteins mediating distinct DNA repair pathways and cell cycle control. In the presence of ATRi and CHK1i, olaparib-induced upregulation of proteins determining cell fate after DNA damage (PARP1, CHK1, c-Abl, Ku70, Ku80, MDM2, and p21) was abrogated in PEO1-OR cells. Overall, the addition of ATRi or CHK1i to olaparib effectively overcomes resistance to PARPi exerting anti-proliferative effect in *BRCA2*^MUT^ olaparib-resistant OC cells and alters expression of DDR-related proteins. These new molecular insights into cellular response to olaparib combined with ATR/CHK1 inhibitors might help improve targeted therapies for olaparib-resistant OC.

## Introduction

Ovarian cancer (OC) is the most lethal gynecologic malignancy, characterized by genomic instability and aberrations in the DNA damage response (DDR)^1–3^. Epithelial OC accounts for about 90% of ovarian tumors and is represented by the most lethal subtype, high-grade serous OC (HGSOC)^4^. Currently, poly(ADP-ribose) polymerase (PARP) inhibition is part of the standard treatment regimen for HGSOC, mainly as maintenance therapy for platinum-sensitive patients with *BRCA1/2* mutations. PARP inhibitors (PARPi) have also shown efficacy in OC patients with other defects in the homologous recombination (HR) repair pathway irrespective of *BRCA1/2* status, supporting their extended clinical use^5^. Olaparib is an approved first-in-class small-molecule inhibitor of PARP1. Blockage of the activity of PARP1 induces accumulating DNA damage and, ultimately, death of OC cancer cells. However, with increasing use of olaparib, many OC patients ultimately develop acquired resistance to the drug and other PARPi treatments, which limits the effectiveness of this therapy^6–8^. Resistance of OC cells to olaparib can arise through a variety of mechanisms, including restoration of DNA repair by HR as the predominant pathway associated with secondary reversion mutations in *BRCA1/2* genes, restoration of replication fork stability, increased PARP1 expression, and other mechanisms^9,10^. Elucidation of alterations in DNA repair pathways beyond HR repair is of major interest in terms of understanding the development of resistance to olaparib in ovarian cancer^11^.

The DDR constitutes a complex network of pathways that coordinate DNA damage sensing and repair, which overlap with cell cycle control and signaling for initiation of apoptosis upon detection of severe lesions. The core DNA repair pathways include direct repair (DR), mismatch repair (MMR), nucleotide excision repair (NER), base excision repair (BER), homologous recombination (HR) repair, and non-homologous end joining (NHEJ), and involve orchestrated cooperation of numerous proteins. The choice of pathway relies on the type of DNA damage, which is repaired by specific individual or coordinated mechanisms^1^. In response to DNA double-strand breaks (DSB), high-fidelity HR or error-prone NHEJ repair is mainly activated. Proteins involved in DDR signaling possess distinct functions as DNA damage sensors, transducers, or effectors. Importantly, defects in one of the pathways can be compensated by the activities of others. However, when the functions of DDR-related proteins are altered, DNA damage cannot be effectively repaired, triggering cell death^2^.

HGSOC is hallmarked by alterations in DDR and genomic instability that are significantly associated with mutations in tumor suppressor p53 and BRCA1/2 proteins^12–14^. Over recent years, knowledge of the DDR process in OC has significantly expanded to inform novel therapeutic strategies^15^. The well-characterized PARPi, olaparib, inhibits the activity of PARP1 involved in sensing DNA single-strand breaks (SSB) as part of a DDR, which eventually leads to repair of accumulating DSBs by HR^16^. Hence, PARP inhibition shows optimal efficacy in the treatment of HR repair-deficient OC cells as an example of a strategy of synthetic lethality between HR deficiency and PARPi^17,18^.

Targeting DDR pathways has been validated as an attractive strategy for enhancing the antitumor activity of PARPi^1,15^. Concurrent inhibition of the ATR/CHK1 pathway is a promising means to restore olaparib activity in some OC types, including cell lines with BRCA1 proficiency and deficiency, as well as HGSOC patients with germline *BRCA1/2* mutations who show progression prior to PARPi therapy^19–21^. The ATR/CHK1 pathway is a critical component of the DDR and involved in regulating cell cycle checkpoints and DNA repair processes in OC cells^2^. Ataxia telangiectasia-mutated and Rad3-related (ATR) kinase functions as a sensor of the presence of SSBs arising from DSBs, stalled replication forks, and NER intermediates. Upon DNA damage and replication stress, ATR transduces the signal through effector proteins including its main target, checkpoint kinase 1 (CHK1), to exert cell cycle control at the G2/M checkpoint in OC^1^. Novel therapeutic opportunities for OC have effectively exploited the ATR/CHK1 pathway. ATR or CHK1 inhibitors can enhance the cytotoxic effects of agents targeting PARP in OC cells by inhibiting HR-mediated DNA repair and promoting the accumulation of DNA damage^9,22^. Preclinical and clinical evidence collectively support the rationale for combining olaparib with inhibitors of the ATR/CHK1 pathway as a therapeutic option. A recent trial exploring a combination of olaparib with the ATR inhibitor (ATRi) ceralasertib in advanced cancers harboring DDR alterations showed high clinical benefit rates in a cohort of *BRCA1/2*-mutated HGSOC patients showing progression with prior PARPi-based regimens^21^. However, further studies are essential to clarify the potential antitumor activity of olaparib combined with inhibitors of the ATR/CHK1 pathway in OC cells harboring various DDR alterations.

Here, we aimed to provide mechanistic insights into the rationale for combining olaparib with inhibitors of the ATR/CHK1 pathway to overcome resistance to PARPi in OC cells with restored BRCA2. Furthermore, we explored the molecular mechanisms underlying the response of OC cells to combination treatments associated with alterations in DDR pathways. Changes in expression of proteins that sense and trigger responses to DNA damage and the pathways by which the response cascade affects effector proteins involved in DDR were comprehensively examined.

## Results

### Inhibitors of the ATR/CHK1 pathway synergistically increase the cytotoxicity of olaparib in resistant OC cells in vitro

**Figure 1:**
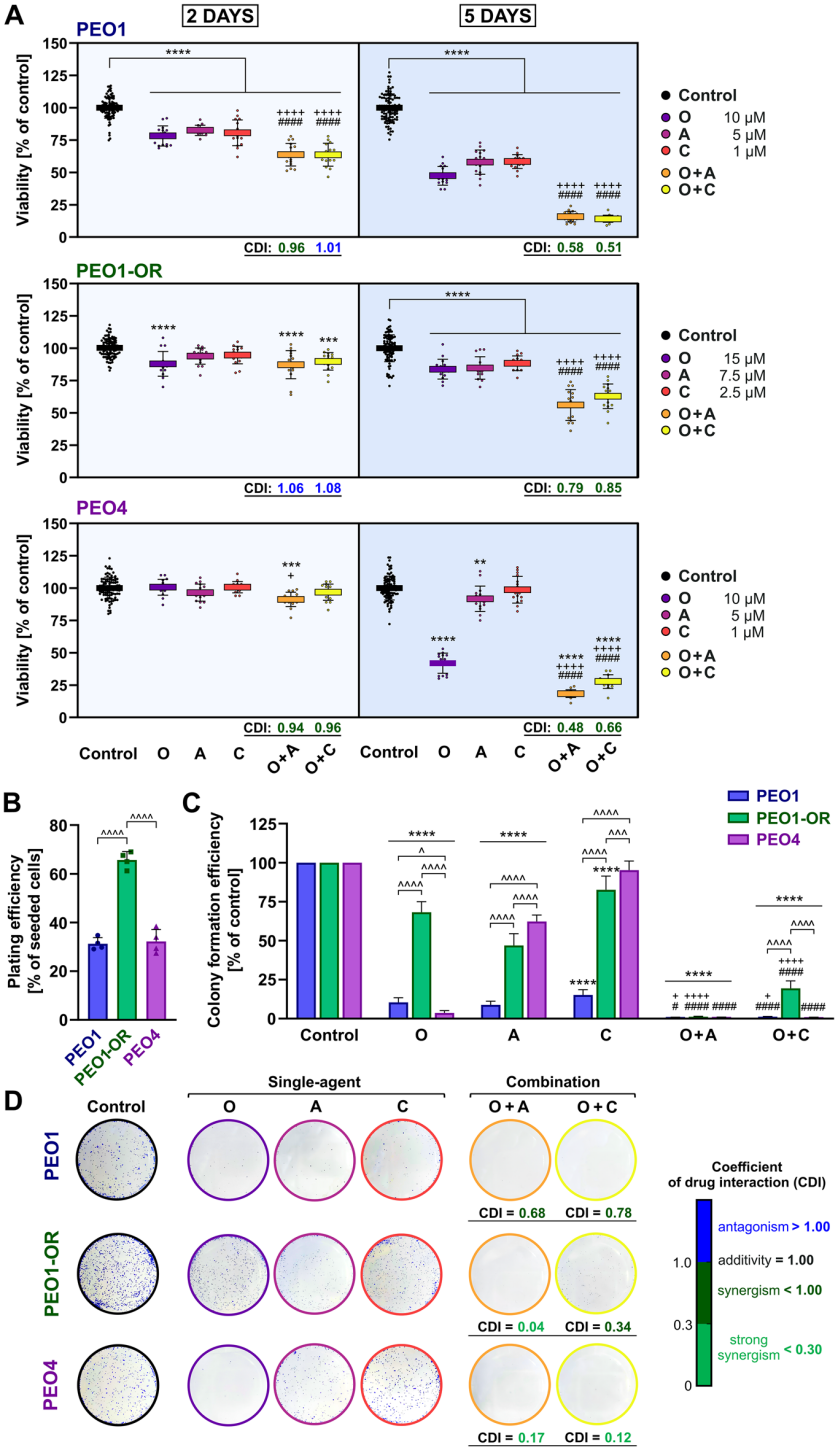
Synergistically reduced viability and clonogenic survival of PEO1-OR ovarian cancer (OC) cells resistant to olaparib in response to treatment with olaparib (O) combined with ATRi (A) or CHK1i (C) relative to each agent alone. (**A**) Cell viability was assessed using the MTT assay. OC cells were treated with the inhibitors (O, A, C) or their combinations (O + A, O + C) at the specified concentrations for 2 and 5 days. Data are presented as mean ± SD (n = 4). Coefficient of drug interaction (CDI) values were calculated to evaluate the effects of combination treatments. (**B**) Determination of plating efficiency of untreated OC cells following plating and incubation of 2000 control cells to form colonies in drug-free medium. Data are presented as mean ± SD (n = 4). (**C**) Clonogenic survival of OC cells was evaluated using a clonogenic assay. OC cells were plated and treated with the inhibitors (O, A, C) or their combinations (O + A, O + C) for 5 days, followed by incubation in drug-free medium for colony formation (7–10 days). Data are presented as mean ± SD (n = 4). Coefficient of drug interaction (CDI) values were calculated to evaluate the interaction effects of the combination treatments. (**D**) Representative images of colonies produced by OC cells in response to treatments. Statistical significance was assessed using ordinary one-way (plating efficiency) or two-way ANOVA (viability, colony formation efficiency) followed by the appropriate multiple comparison tests: ^*^*^*p* < 0.05, ^*^^^*^*p* < 0.001, ^*^^^^*^*p* < 0.0001 (comparison between cell lines); **p < 0.01, ***p < 0.001, ****p < 0.0001 (treatment vs. control); ^+^*p* < 0.05, ^++++^*p* < 0.0001 (olaparib vs. combination with ATRi or CHK1i); ^*####*^*p* < 0.0001 (ATRi or CHK1i vs. respective combinations with olaparib).

We initially examined the hypothesis that addition of ATR/CHK1 pathway inhibitors could enhance olaparib cytotoxicity in olaparib-resistant OC cells. The cytotoxic effect of olaparib in combination with ATR/CHK1 inhibitors (ATRi/CHK1i) was evaluated in HGSOC cells treated for 2 and 5 days with 10 μM olaparib, 5 μM ATRi, and 1 μM CHK1i, in addition to PEO1-OR (olaparib-resistant) cells treated with 15 μM olaparib, 7.5 μM ATRi, and 2.5 μM CHK1i via the MTT assay (Fig. 1). Coefficient of drug interaction (CDI) values were calculated to determine whether the effect of combined treatment with olaparib and inhibitors was synergistic, as described in the “Materials and methods” (“MTT cell viability assay” section).

Both combination treatments exhibited nearly additive to slightly synergistic cytotoxic effects (CDI = 0.94–1.01) after 2 days of incubation in olaparib-sensitive OC cells (Fig. 1A). PEO1 cell viability was significantly reduced to ~ 64% in response to both combinations compared to each agent alone after 2 days. After 5 days of incubation, both ATRi and CHK1i combinations with olaparib induced a favorable synergistic reduction in PEO1 and PEO4 cell viability (CDI = 0.48–0.66) (Fig. 1A). Clearly, the addition of ATRi or CHK1i to olaparib markedly increased the cytotoxic effects of the individual agents, leading to a significant decrease in the metabolic activity of PEO1 (15.8% and 14.1%) and PEO4 (18.4% and 27.7%).

The effects of treatment of PEO1-OR cells with inhibitors at the above concentrations ranged from slightly antagonistic and nearly additive (CDI = 1.02 and 1.09) after 2 days to slightly synergistic (CDI = 0.90 and 0.93) after 5 days (Fig. S1). A satisfactory synergistic effect of olaparib with ATRi or CHK1i (CDI = 0.79 and 0.85, respectively) was achieved at higher concentrations of tested inhibitors after 5 days of incubation (Fig. 1A). Irrespective of the time of incubation, 15 μM olaparib alone exerted only a slight cytotoxic activity in PEO1-OR cells, as evident from no significant differences in cell viability between 2 and 5 days of treatment (87.9% and 83.8%, *p* = 0.72). Compared to each agent alone, combination of ATRi and CHK1i with olaparib induced a significant decrease in metabolic activity of PEO1-OR cells to about 56% and 63%, respectively. The results collectively demonstrated that after 5 days of treatment, ATR and CHK1 inhibitors synergistically enhanced the slightly cytotoxic effect of olaparib on the olaparib-resistant cell line.

### Olaparib exerts synergistic suppressive effects with inhibitors of the ATR/CHK1 pathway on clonogenic survival in olaparib-resistant OC cells

Survival of OC cell lines in response to treatments with the inhibitors alone or in combination with olaparib was investigated via the clonogenic assay (Fig. 1B–D). Colony formation of seeded OC cells treated for 5 days was examined. The results showed that untreated PEO1-OR cells exhibited two times higher plating efficiency than olaparib-sensitive cells (Fig. 1B). Importantly, both combination treatments synergistically decreased the clonogenic survival of olaparib-sensitive and -resistant OC cell lines compared to either agent alone (Fig. 1C). Interestingly, although PEO1-OR cells were significantly less sensitive to olaparib alone than PEO1 (6.5 times) and PEO4 cells (18.5 times), the addition of ATRi markedly decreased clonogenic survival, resulting in almost complete inhibition of the ability to form colonies in both olaparib-sensitive and olaparib-resistant cell lines. The colony-forming ability of all OC cell lines was also synergistically reduced in response to the combined treatment with olaparib and CHK1i. However, in the presence of olaparib, PEO1-OR cells displayed a more significant response to ATRi than CHK1i, as indicated by the higher synergistic interactions between olaparib and ATRi (CDI = 0.04) than CHK1i (CDI = 0.34) (Fig. 1C). The observed inhibitory effects of the drug combinations on clonogenic survival are in line with synergistically decreased viability of PEO1-OR cells after 5 days of incubation, as determined with the MTT assay (Fig. 1A). Our collective data indicate that addition of ATR/CHK1 kinase inhibitors to olaparib exerts a synergistic increase in cytotoxicity compared to each agent alone in PEO1-OR olaparib-resistant cells, leading to significantly decreased cell viability and clonogenic survival.

### Olaparib acts synergistically with inhibitors of the ATR/CHK1 pathway to enhance activity of caspase-3 and -7 in mediating apoptosis in olaparib-resistant cells

**Figure 2:**
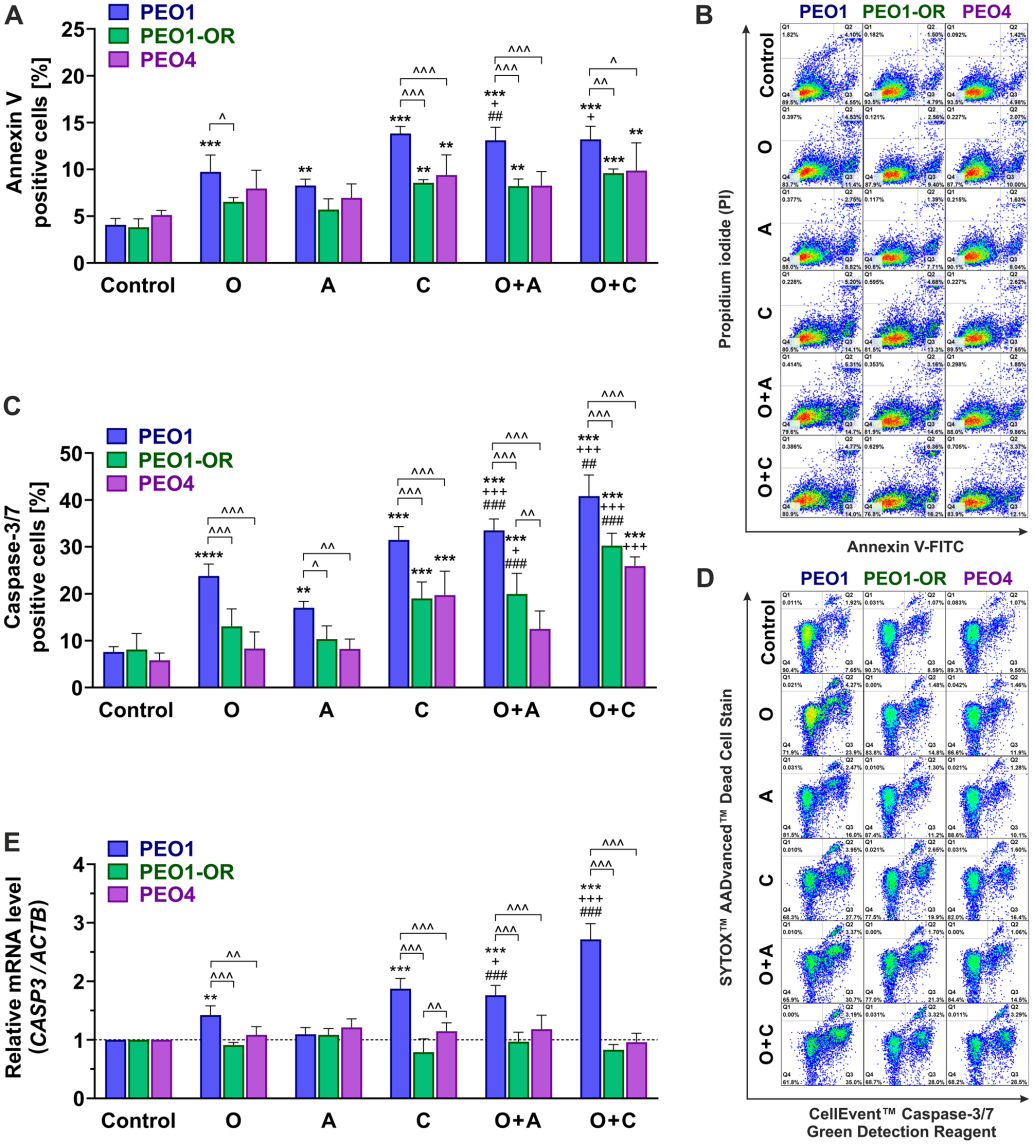
Induction of apoptosis and synergistic activation of caspase-3 and -7 in PEO1-OR ovarian cancer (OC) cells in response to olaparib (O) combined with ATRi (A) or CHK1i (C) compared to each agent alone. OC cells were treated with the inhibitors (O, A, C) or their combinations (O + A, O + C) for 2 days. (**A**) Apoptosis was examined via dual staining with annexin V-FITC and PI, quantified with flow cytometry, and presented as a percentage of annexin V-positive (apoptotic) relative to untreated control cells. Data are presented as mean ± SD (n = 3). (**B**) Representative dot plots of annexin V-FITC- and PI-stained OC cells with the indicated percentages of non-viable cells (Q1), necrotic cells (Q2), apoptotic cells (Q3), and viable cells (Q4). (**C**) Activation of caspase-3 and caspase-7 in apoptotic cells examined via dual staining and flow cytometry using a CellEvent™ Caspase-3/7 Green Flow Cytometry Assay Kit, expressed as a percentage of caspase-3/7-positive relative to untreated control cells. Data are presented as mean ± SD (n = 3). (**D**) Representative dot plots of a two-parameter apoptosis assay for detection of activated caspase-3/7 (CellEvent™ Caspase-3/7 Green Detection Reagent) and discrimination between live and dead cells (SYTOX™ AADvanced™ Dead Cell Stain) with the indicated percentages of necrotic cells (Q2), viable cells (Q3), and apoptotic cells with activated caspase-3/7 (Q4). (**E**) qRT-PCR analysis of caspase-3 mRNA. Relative mRNA expression was calculated using the 2^−∆∆Ct^ method and β-actin (*ACTB*) as a reference gene. Data are presented as mean ± SD (n = 4). Statistical significance was assessed using two-way ANOVA, followed by Tukey’s multiple comparison test: ^*^*^*p* < 0.05, ^*^^*^*p* < 0.01, ^*^^^*^*p* < 0.001 (comparison between cell lines); **p < 0.01, ***p < 0.001, ****p < 0.0001 (treatment vs. control); ^+^*p* < 0.05, ^+++^*p* < 0.001 (olaparib vs. combination with ATRi or CHK1i); ^*##*^*p* < 0.01, ^*###*^*p* < 0.001 (ATRi or CHK1i vs. respective combinations with olaparib).

We examined the hypothesis that combined treatment with inhibitors of the ATR/CHK1 pathway and olaparib could enhance apoptosis in OC cells with acquired resistance to olaparib. Firstly, annexin V-FITC/PI dual staining was performed to examine the effects of combination treatments on phosphatidylserine externalization as a marker of apoptosis in OC cells after 2 days (Fig. 2A,B).

Treatment of PEO1 cells with olaparib alone caused a significant (2.4-fold) increase in annexin V-positive cells compared with untreated cells (from 4.1% to 9.7% apoptotic cells), which was further synergistically augmented with the addition of ATRi (13.1% apoptotic cells) (Fig. 2A). In contrast, in PEO1-OR cells, olaparib or ATRi alone induced a non-significant change in the percentage of apoptotic cells (from 3.8 to 6.5% and 5.7%, respectively). Importantly, concurrent incubation with olaparib and ATRi induced a significant increase (2.2-fold) in the apoptotic cell content relative to untreated PEO1-OR cells. CHK1i alone and in combination with olaparib significantly elevated phosphatidyl externalization in PEO1-OR cells. However, the observed effect was not synergistic, with similar levels of increase in annexin V-positive cells of 8.6% and 9.6%, respectively (Fig. 2A). PEO4 cells exhibited similar minor changes in phosphatidylserine externalization as PEO1-OR cells after incubation with olaparib or ATRi alone. The most significant (almost 2.0-fold) increase in annexin V-positive cells was observed in response to a combination of olaparib and CHK1i relative to the control group (from 5.1 to 9.9%). However, this effect was similar to that of CHK1i alone (Fig. 2A).

To confirm the apoptosis-inducing effect of the combination treatment, we further investigated activation of caspase-3 and -7 in OC cells as an early event during apoptotic death and further explored whether the interaction effect between inhibitors is synergistic at the early stages at the 2-day stage (Fig. 2C,D). Olaparib alone caused significant (3.1-fold) elevation in the percentage of caspase-3 and -7-positive cells in the PEO1 cell line only (7.6% to 23.8%). Combination treatments led to a significant synergistic increase in caspase-3 and -7 activities in both PEO1 and PEO1-OR cells, although PEO1 cells were considerably more sensitive. In the olaparib-resistant cell line, olaparib and ATRi alone did not affect the activities of caspase-3 and -7. Notably, the percentage of caspase-3- and -7-positive cells increased from 13.1% in olaparib-treated cells by 1.5-fold and 2.3-fold in the presence of ATRi and CHK1i, respectively (Fig. 2C), indicating a synergistic effect. Interestingly, combination of olaparib with ATRi had a non-significant effect on phosphatidylserine externalization and caspase-3 and -7 activities in olaparib-sensitive PEO4 cells (Fig. 2A–C), which correlated with a negligible decrease in viability of this cell line in response to combination treatments after 2 days of incubation (Fig. 1A).

Caspase-3 mRNA was significantly increased in olaparib-sensitive PEO1 cells in the presence of olaparib (Fig. 2E). Moreover, the effect exerted by olaparib was synergistically augmented by ATRi and CHK1i in PEO1 cells (1.2-fold and 1.9-fold compared with olaparib alone), in line with increased activity of caspase-3 and -7, as determined with flow cytometry (Fig. 2E). In contrast, olaparib-resistant PEO1-OR cells showed no significant changes in *CASP3* levels in response to either individual inhibitors or their combinations after 2 days of incubation (Fig. 2E).

Altogether, olaparib combined with inhibitors of the ATR/CHK1 pathway exerted a slight synergistic proapoptotic effect in olaparib-resistant PEO1-OR cells after 2 days of incubation, accompanied by increased caspase-3 and -7 activities and phosphatidylserine externalization. The synergistic proapoptotic activity of the combined inhibitors in PEO1-OR cells was significantly increased with prolonged exposure up to 5 days.

### Combination of olaparib with ATR/CHK1 inhibitors dysregulates protein expression in the DNA damage response pathway in the olaparib-resistant OC cell line

**Figure 3:**
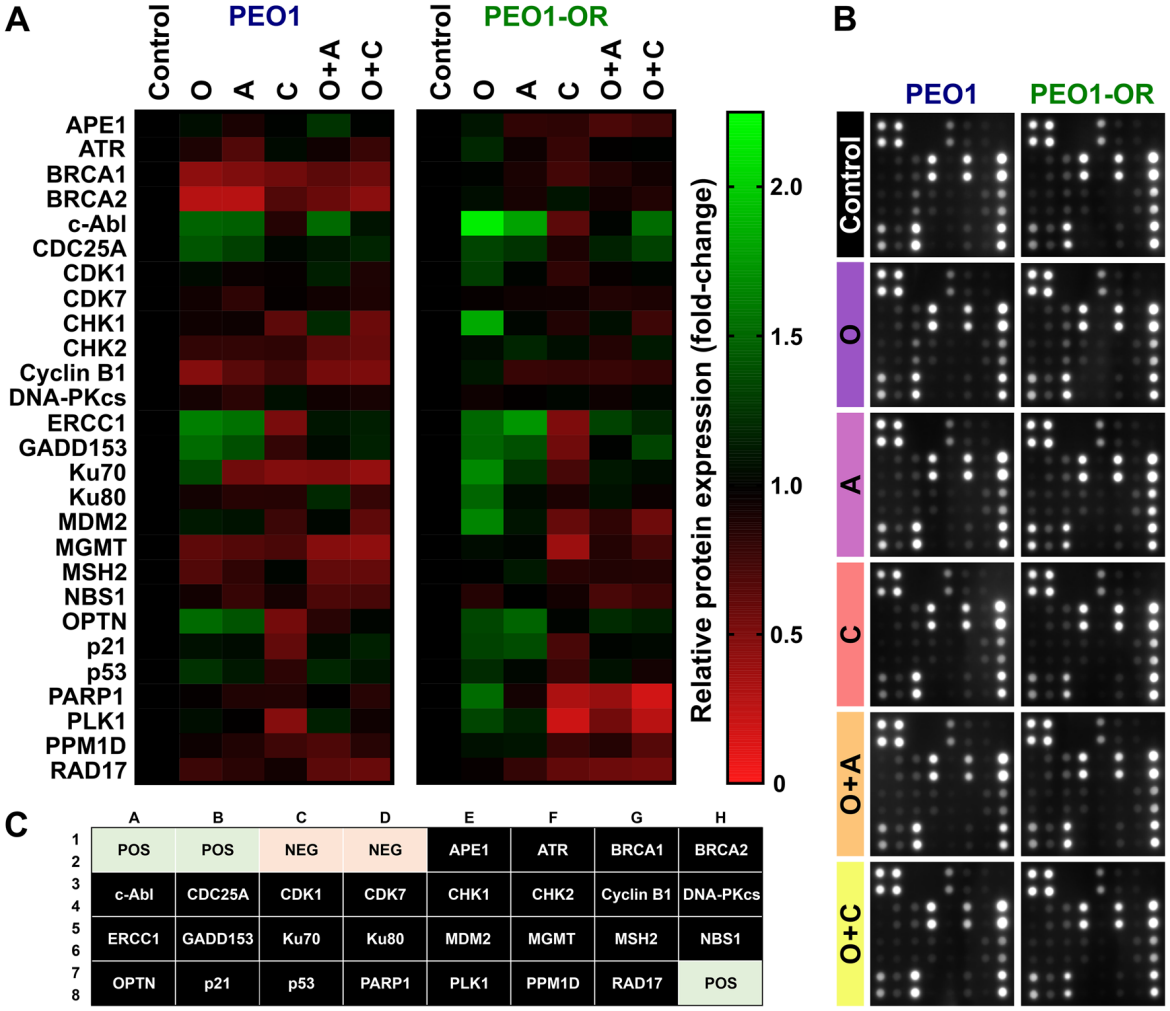
Expression profiles of DNA damage response (DDR)-related proteins in olaparib-sensitive and -resistant OC cell lines in response to olaparib (O) combined with ATRi (A) or CHK1i (C). OC cells were treated with the inhibitors (O, A, C) or their combinations (O + A, O + C) for 2 days and whole-cell lysates used for analysis of 27 DDR-related proteins via antibody arrays. (**A**) Heat map representing relative expression of DDR-associated proteins in OC cell lines treated with inhibitors (n = 4). The gradient scale represents normalized expression values. (**B**) Original representative images of antibody arrays. (**C**) Array map showing the locations of individual antigen-specific antibody spots printed in duplicate. *POS* positive control spots used for data normalization between arrays, *NEG* negative control spots used to measure the baseline signal.

**Figure 4:**
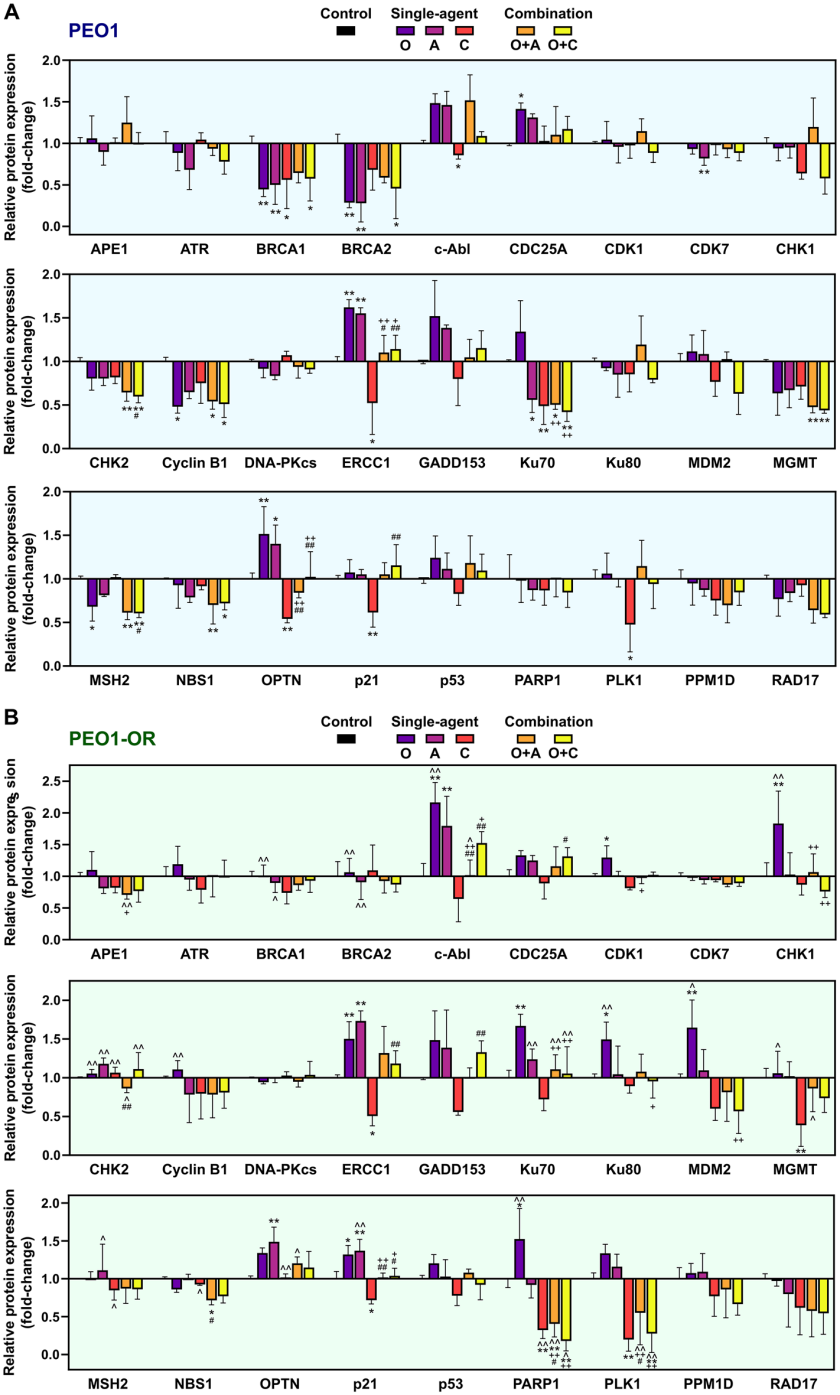
Olaparib (O) combined with ATRi (A) or CHK1i (C) dysregulates expression of DNA damage response (DDR)-related proteins in OC cells. Semi-quantitative analysis of DDR-associated proteins expression using antibody microarray in PEO1 (**A**) and PEO1-OR (**B**) cells treated with the inhibitors (O, A, C) or their combinations (O + A, O + C) for 2 days. Data are expressed as mean ± SD (n = 4) relative to untreated control cells. Statistical significance was assessed using two-way ANOVA, followed by Tukey’s multiple comparison test: ^*^*^*p* < 0.05, ^*^^*^*p* < 0.01 (comparison between cell lines); *p < 0.05, **p < 0.01 (treatment vs. control); ^+^*p* < 0.05, ^++^*p* < 0.01 (olaparib vs. combination with ATRi or CHK1i); ^*#*^*p* < 0.05, ^*##*^*p* < 0.01 (ATRi or CHK1i vs. respective combinations with olaparib).

The DNA-damaging activity of olaparib plays a critical role in OC cell cytotoxicity. To establish whether restoration of olaparib cytotoxicity by inhibitors of the ATR/CHK1 pathway in resistant cells is associated with DDR-related proteins, we compared the expression profiles of 27 proteins involved in DDR in both PEO1 and PEO1-OR cells using antibody arrays (Figs. 3, 4).

Firstly, we focused on response to olaparib alone in PEO1-OR cells with markedly diminished sensitivity to this PARPi compared with PEO1 cells. A total of 7 and 9 proteins displayed significant differences in response to olaparib in PEO1 and PEO1-OR cells, respectively (Figs. 3, 4). Moreover, among all the DDR-related proteins examined, expression levels of BRCA1, BRCA2, c-Abl, CHK1, CHK2, cyclin B1, Ku80, MDM2, MGMT, and PARP1 were markedly different between the two cell lines incubated with olaparib (Figs. 3, 4).

Comparative semi-quantitative analysis of protein expression revealed that treatment with olaparib caused significant downregulation of proteins involved in promoting HR repair (BRCA1 by 2.3-fold, BRCA2 by 3.5-fold) and cell cycle control (cyclin B1 by 2.1-fold) in PEO1 cells (Fig. 4A). In contrast, treatment with olaparib had no significant effects on expression of these proteins in PEO1-OR cells (Fig. 4B). However, olaparib induced a marked increase in expression of c-Abl (by 2.2-fold), CHK1 (by 1.8-fold), MDM2 (by 1.7-fold), Ku80 (by 1.5-fold), p21 (by 1.3-fold) and PARP1 (by 1.5-fold) in PEO1-OR cells, which was not observed in PEO1 cells (Fig. 4).

**Figure 5:**
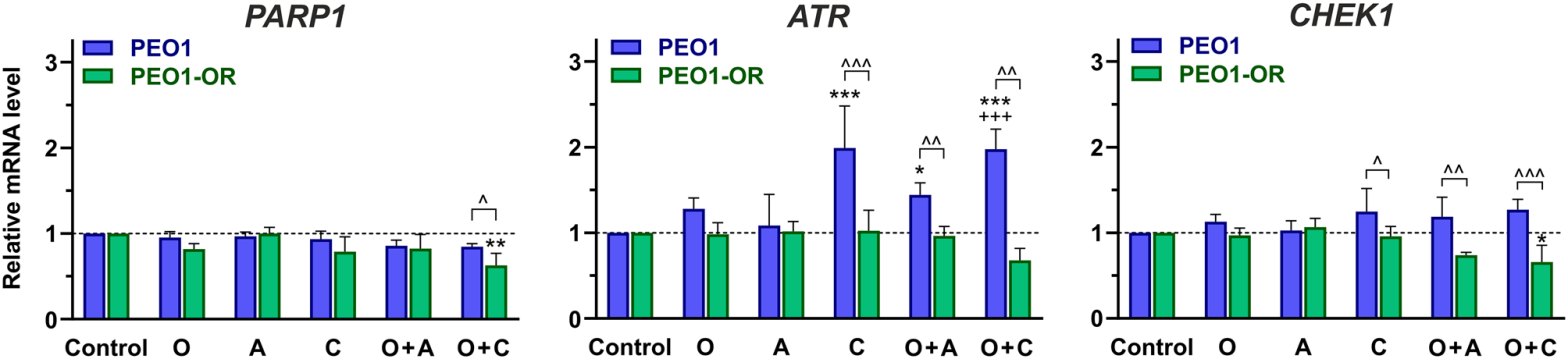
Olaparib combined with the CHK1 inhibitor decreases mRNA levels of *PARP1* and *CHEK1* in olaparib-resistant PEO1-OR cells. OC cells were treated with the inhibitors (O, A, C) or their combinations (O + A, O + C) for 2 days and mRNA levels determined via real-time qPCR. Data were normalized to β-actin and expressed relative to untreated cells as mean ± SD (n = 4). Statistical significance was assessed using two-way ANOVA, followed by Tukey’s multiple comparison test: ^*^*^*p* < 0.05, ^*^^*^*p* < 0.01, ^*^^^*^*p* < 0.001 (comparison between cell lines); *p < 0.05, **p < 0.01, ***p < 0.001 (treatment vs. control); ^+++^*p* < 0.001 (olaparib vs. combination with ATRi or CHK1i).

Comparison of protein levels after treatment with each agent alone and their combinations revealed significant changes in expression of seven proteins in both PEO1 (CHK2, ERCC1, MGMT, NBS1, OPTN, p21, PLK1) and PEO1-OR (c-Abl, ERCC1, MGMT, OPTN, p21, PARP1, PLK1) cell lines (Fig. 4). Expression of proteins directly targeted by the inhibitors, i.e., PARP1, ATR, and CHK1, was not significantly changed in PEO1 cells in response to treatments (Fig. 4A). These findings were confirmed at mRNA level for *PARP1* and *CHEK1* (Fig. 5). In contrast to PEO1 cells, co-treatment with ATRi and olaparib caused a 2.5-fold decrease in PARP1 expression in PEO1-OR compared to untreated cells (Fig. 4B). However, no changes in *PARP1* mRNA levels were observed in response to the combination treatment in PEO1-OR cells (Fig. 5). Moreover, combination of olaparib with either ATRi or CHK1i abolished olaparib-induced upregulation of CHK1 in PEO1-OR cells (Fig. 4B). Distinct from DDR antibody array data, a significant decrease in *CHEK1* mRNA was observed in PEO1-OR cells in response to concurrent treatment with olaparib and CHK1i (Fig. 5).

Regarding effects of the combination treatments on proteins involved in HR repair, no significant changes were observed in BRCA1 and BRCA2 representing key mediators of this pathway in PEO1-OR cells (Fig. 4B). However, inhibition of the ATR/CHK1 pathway abolished olaparib-induced upregulation of c-Abl kinase that acts as a positive regulator of HR repair. Analysis of NHEJ pathway components in PEO1-OR cells showed that protein expression of Ku70 and Ku80, but not DNA-PKcs, was significantly increased by 1.7-fold and 1.5-fold after treatment with olaparib alone. This effect was abolished with concurrent inhibition of the ATR/CHK1 pathway (Fig. 4B). In contrast, PEO1 cells showed no changes in expression of NHEJ-promoting proteins treated with either agent alone or in combination (Fig. 4A). In the case of proteins involved in other DDR pathways, the addition of either ATRi or CHK1i to olaparib restored basal levels of the NER-promoting endonuclease ERCC1 in both PEO1 and PEO1-OR cell lines (Fig. 4). Moreover, downregulation of methyltransferase MGMT, a key enzyme in the DR pathway, observed in PEO1 cells in response to olaparib combined with inhibitors of the ATR/CHK1 pathway, was abrogated in PEO1-OR cells.

Antibody array data also revealed changes in expression patterns of proteins involved in the G2/M phase transition downstream of the ATR/CHK1 pathway. We observed significant downregulation of cyclin B1 in PEO1 cells treated with both olaparib alone and combined with ATRi or CHK1i, which induced a similar decrease in cyclin B1 levels by about 2.1 times compared to untreated cells (Fig. 4A). In contrast to olaparib-sensitive cells, in PEO1-OR cells, expression of cyclin B1 remained unchanged irrespective of the treatment. However, both combinations restored basal levels of p21 and markedly decreased PLK1 expression in PEO1-OR cells by about 1.8-fold (with ATRi) and 3.6-fold (with CHK1i) (Fig. 4B).

## Discussion

Olaparib exhibits clinical efficacy in OC patients, but PARPi resistance remains a major challenge in long-term treatment. Targeting of the ATR/CHK1 pathway with small-molecule inhibitors sensitizes OC to olaparib with distinct genetic alterations related to DDR pathways^19–21,23^. Importantly, ATR^21^ or CKH1^24^ kinase inhibitors combined with olaparib show favorable preliminary clinical activity in PARPi-resistant HGSOC patients. However, some of them progress after combination treatments^24^ or do not achieve effective response^25^, supposedly due to unknown factors that are yet to be established. In the present study, we explored combining olaparib with ATR/CHK1 pathway inhibitors in the previously developed PEO1-OR cell line with acquired resistance to olaparib and restored BRCA2^9^. Although *BRCA2* reversion mutations occur in the minority of OC patients treated with PARPi^26,27^, they tend to accumulate upon progression^27^ and represent a well-established mechanism of olaparib resistance. Importantly, our cell line serves as a valuable model of olaparib resistance, mirroring reversion mutations in *BRCA2* in PARPi-treated HGSOC patients^28^. Here, we addressed mechanisms associated with overcoming resistance to olaparib concerning changes in the expression of DDR-related proteins in PEO1-OR cells.

Previously we revealed differences in the responses of olaparib-sensitive and -resistant cell lines to ATR/CHK1 pathway inhibitors in the presence of PARPi at non-toxic concentrations for 2 days for PEO1-OR cells^29^. Here, we focused on combating resistance to olaparib via combined treatment with olaparib, ATRi, and CHK1i exerting synergistic antitumor effects. Firstly, we confirmed that inhibition of the ATR/CHK1 pathway synergistically enhanced olaparib cytotoxicity in PEO1-OR cells. Olaparib alone modestly affected the viability of PEO1-OR cells after 2 and 5 days, but combination treatment for 5 days decreased the colony-forming ability of PEO1-OR cells to similar levels as olaparib-sensitive cells, confirming durable responses. Supposedly, more than a single round of replication is required to induce significant antitumor actions in olaparib-resistant cells, affirming olaparib’s need for multiple rounds of cell division to exert its cytotoxic activity^30^. The observations are in line with earlier reports^19,23^ and endorse overcoming resistance to olaparib by adding ATRi or CHK1i to PARPi.

Apoptotic changes in OC cells were additionally examined. In PEO1 cells, PS externalization, caspase-3/7 activity, and *CASP3* mRNA levels synergistically increased with combination treatments compared to single-agent inhibitors within 2 days. Adding ATRi or CHK1i synergistically elevated caspase activity in PEO1-OR cells irrespective of *CASP3* transcriptional level. PS externalization tended to increase in PEO1-OR cells after 2 days in response to olaparib when combined with ATRi or CHK1i. These early membrane asymmetry changes aligned with a synergistic decrease in cell survival and growth after 5 days of treatment. Our results suggest differential control of PS exposure associated with apoptosis in olaparib-sensitive and -resistant cells. Variability in surface PS exposure within cancer types, correlating with treatment susceptibility^31,32^, further supports our observations. A study by Kim et al.^23^ revealed increased PS externalization in OC olaparib-resistant cell lines after 3 days of olaparib combined with ATRi. Olaparib reportedly synergizes with arsenic trioxide in platinum-resistant OC cells to increase cleavage of caspase-3 and promote PS externalization within 2 days^33^. Significantly, all examined OC cells showed synergistic elevation of caspase-3/7 activity with combination treatments, indicating reduced cell growth primarily through caspase-mediated apoptosis. Our data provide evidence that relatively short incubation with combined inhibitors holds promising proapoptotic activity in PEO1-OR cells.

OC cells exhibit various changes in DDR pathways, particularly HR deficiency, which are exploited by PARPi treatments^11,17,34^. PARP1 inhibits DNA DSB end resection antagonizing HR and promoting NHEJ^35^, and regulates SSB repair through NER and BER^36^. Inhibition with olaparib may affect DDR pathways and drive response to PARPi beyond HR effectiveness in OC cells^37^. Evidence from OC indicates that concurrent downregulation of HR and either NER or MMR pathways positively correlates with sensitivity to olaparib^34^. Over recent years, targeting the DDR pathway has become a key strategy to overcome resistance to PARPi^1,34^. ATR/CHK1 inhibition shows promise in the resensitization to olaparib, especially in *BRCA1*^MUT^ OC, but challenges exist in *BRCA2*^MUT^ tumors. Further investigation of the utility of ATR/CHK1 pathway inhibitors in the resensitization of OC to olaparib is necessary.

**Figure 6:**
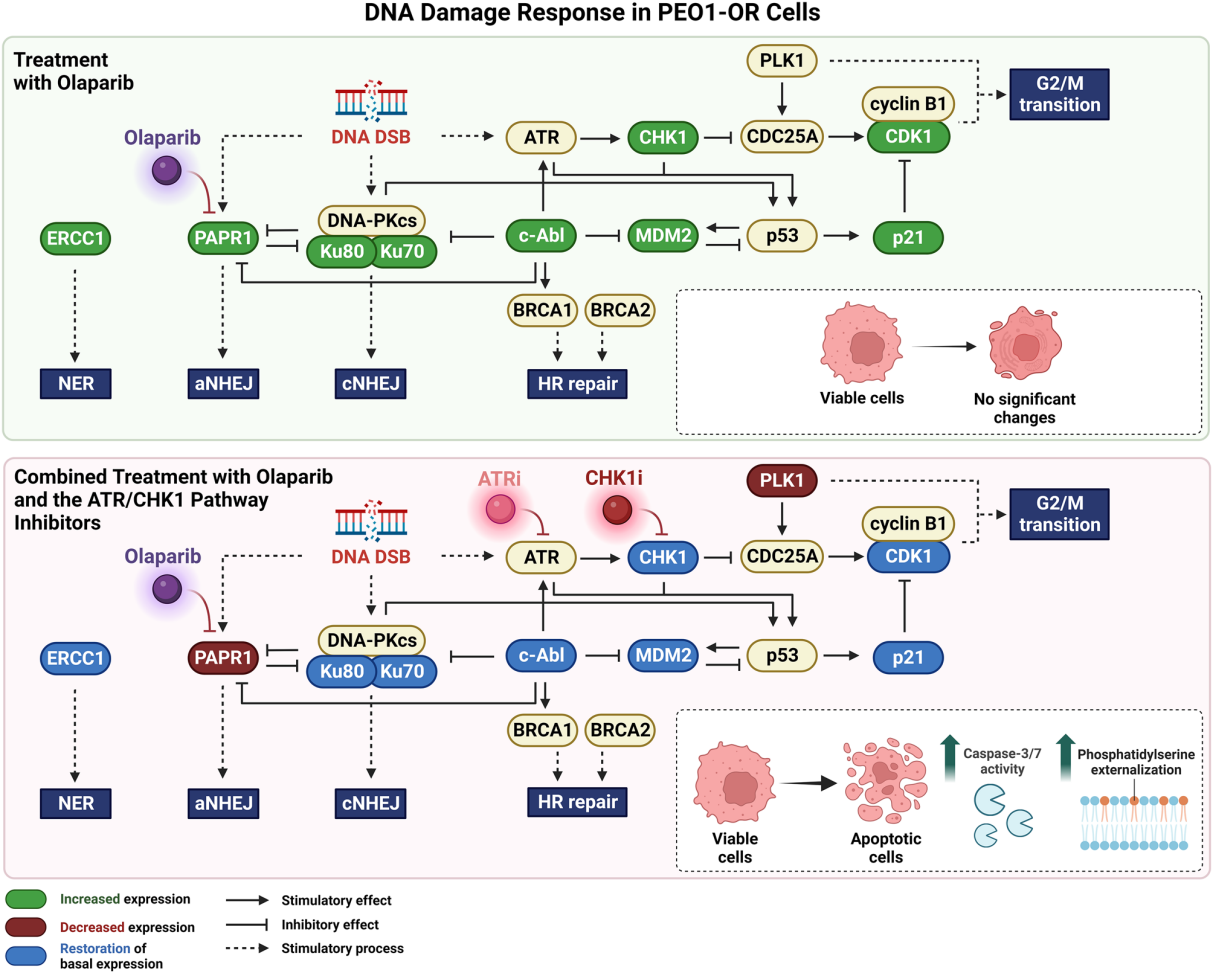
Overview of alterations in expression of proteins involved in DDR induced by inhibitors of the ATR/CHK1 pathway that potentially contribute to overcoming resistance to olaparib in PEO1-OR cells. *aNHEJ* alternative non-homologous end joining, *cNHEJ* canonical non-homologous end joining, *DSB* double-strand break, *HR* homologous recombination, *NER* nucleotide excision repair.

In this study, we provide evidence that a combination of olaparib with the ATR/CHK1 pathway inhibitors induces cumulative alterations in the expression of DDR-related proteins in the olaparib-resistant OC cells (Fig. 6). Olaparib-induced downregulation of BRCA1 and BRCA2 in olaparib-sensitive PEO1 cells was abrogated in PEO1-OR cells. Olaparib combined with CHK1i suppressed expression of BRCA1 and BRCA2 in PEO1 cells similarly to olaparib alone. However, despite the effective treatment of PEO1-OR cells with olaparib and ATR/CHK1 pathway inhibitors, no significant impact on BRCA1 and BRCA2 levels was observed (Fig. 6), indicating different HR-related DDR responses to combinations.

NHEJ repairs DNA DSBs throughout the cell cycle, with Ku70, Ku80, and DNA-PKcs regulating the canonical pathway^2^. Alternatively, DSBs are recognized by PARP1, which removes Ku70/80 complexes from damage sites exclusively during the S/G2 phase and promotes the alternative NHEJ pathway^38^ (Fig. 6). The study by McCormick et al.^39^ provided evidence that NHEJ is defective in 40% of ex vivo primary cultures of epithelial OC. Downregulation of Ku70, Ku80, and DNA-PKcs may promote resistance to the PARPi rucaparib, irrespective of HR repair status^39^. Olaparib-induced Ku70 and Ku80 overexpression in PEO1-OR cells was reversed by ATRi or CHK1i co-treatment. Simultaneously, combination treatments downregulated PARP1 in PEO1-OR. Interestingly, previous studies by our group have shown that the combination of olaparib with inhibitors of the ATR/CHK1 pathway decreases the expression of NHEJ-promoting 53BP1^29^.

In response to DNA damage, c-Abl tyrosine kinase mediates phosphorylation of DDR-related substrates, including proteins involved in repair mechanisms (ATR, BRCA1, PARP1, DNA-PKcs), cell cycle regulation, and apoptosis (p53, MDM2). c-Abl has been implicated in promoting DNA repair or proapoptotic signaling under unrepairable DNA damage^40^. For instance, phosphorylation of MDM2 by c-Abl negatively regulates the MDM2-dependent inhibitory effect on the tumor suppressor p53 (Fig. 6)^40^. Importantly, c-Abl overexpression was identified in OC specimens^41,42^, but studies on c-Abl’s role in the DDR of olaparib-resistant OC cells are lacking. Our work revealed olaparib-induced upregulation of c-Abl and MDM2 in PEO1-OR cells, neutralized upon the addition of ATRi or CHK1i. c-Abl upregulation may contribute to decreased sensitivity to olaparib by activating alternative DNA repair pathways that compensate for loss of PARP activity. Indeed, overexpression of MDM2 is linked to increased survival, therapeutic resistance, and metastasis in OC^43,44^. Recent findings indicate that MDM2 inhibition enhances cisplatin’s antitumor activity in platinum-resistant OC cells^45^. We propose that c-Abl-dependent effects on MDM2 and PARP1 may be abolished after combination treatments, and associated with overcoming resistance to olaparib. Moreover, upon DNA damage ATR-mediated phosphorylation inhibits c-Abl substrate MDM2^46^. In our study, olaparib-induced upregulation of c-Abl, MDM2, and CHK1 in PEO1-OR cells was abolished upon co-treatment with ATRi or CHK1i. This suggests that the reliance of olaparib-resistant OC cells on c-Abl-dependent DDR machinery plays an important role in ATR/CHK1 inhibitor-mediated suppression of resistance. Indeed, the analysis of c-Abl-mediated downstream events confirmed that combinations induced apoptosis in PEO1-OR cells.

Olaparib triggers DNA damage-induced G2/M checkpoint arrest and promotes apoptosis in sensitive OC cells^23,47^. We previously demonstrated that PEO1-OR cells bypass G2/M arrest induced by PARPi in olaparib-sensitive cells^29^. Here, we focused on changes in expression of the proteins involved in the G2/M transition (CDC25A, CDK1, CHK1, CHK2, cyclin B1, p21, PLK1). Olaparib downregulated cyclin B1 only in PEO1 cells, indicating decreased G2/M transition, consistent with previous results^29^. This decrease in cyclin B1 levels was abrogated in PEO1-OR cells, while simultaneous upregulation of p21 and CHK1 was observed, which could promote G2/M arrest and reflect an effective response to olaparib^29^. Activation of PLK1 via CHK1 upon DNA damage promotes HR-mediated DSB repair^48^. Moreover, in the absence of PARP1, recruitment of PLK1 to DSBs may be abolished^48^. In our study, combination treatment decreased the levels of PLK1 and PARP1 with concurrent normalization of CHK1 expression to basal levels in PEO1-OR cells, suggesting aberrations in HR repair.

Analysis of the DNA damage sensors provided further insights into variations in DDR among OC cells. Recent studies suggest that the DR-associated DNA damage sensor MGMT promotes chemoresistance, and its inhibition can sensitize OC to histone deacetylase inhibitors^49^. Large-scale screening has revealed that alterations in *MSH2*, a DNA damage recognizer, are extremely rare in OC patients^50^. Data from the current study showed that expression of MSH2 and MGMT is suppressed in PEO1 cells in response to olaparib alone or combined with ATR/CHK1 inhibitors, suggesting deficiencies in DR and MMR pathways in olaparib-sensitive cells. However, PEO1-OR cells appear less susceptible to changes in DNA damage sensors involved in DR and MMR, indicating that alternative DDR pathways play a minor role in the resensitization of OC to olaparib.

DDR inhibitor-based combination therapies hold promise for OC treatment due to tumor susceptibility to DNA damage and reliance on response mechanisms to proliferate^1,51^. Recent studies link DDR signatures with response to chemotherapy in OC patients-derived tumor cells^12^. Dysregulation of DDR-related proteins in OC cells may be critical in selecting predictive biomarkers of treatment response and unraveling the connection between DDR and olaparib resistance. Overall, alterations in DDR-related proteins induced by the concurrent use of olaparib and ATR/CHK1 inhibitors in PEO1-OR cells show promise in postponing or preventing drug resistance in OC cells. Yet, further research is required to establish whether long-term resensitization of OC cells to olaparib combined with ATR/CHK1 pathway inhibitors is achievable.

Notably, our confirmation of overcoming olaparib resistance has been verified in one OC cell line with acquired resistance to olaparib, which limits the finding of our study to the model with *BRCA2* reversion mutation. Nonetheless, the strength of this model lies in the thorough characterization of the PEO1-OR cell line and the investigation of its response to non-toxic concentrations of PARPi in the presence of ATR/CHK1 pathway inhibitors^9^. Despite the potential weaknesses, we clearly showed that resistance to olaparib can be overcome with ATR/CHK1 inhibitors in OC cells.

In conclusion, ATR/CHK1 inhibitors effectively resensitize OC cells with restored BRCA2 to olaparib in vitro, inducing apoptosis through synergistic activation of caspase-3/7. Concurrent treatment with olaparib and ATR/CHK1 pathway inhibitors induces dysregulation of proteins involved in the DDR pathway as well as the G2/M checkpoint during cell cycle control. The collective findings provide novel insights into the molecular mechanisms by which inhibitors of the ATR/CHK1 pathway act synergistically with olaparib to enhance cytotoxicity, supporting the utility of this strategy as a promising treatment for OC with restored *BRCA2*.

## Materials and methods

### Materials

PARPi (O, olaparib, AZD2281) was purchased from Selleck Chemicals (Houston, TX, USA). ATRi (A, ceralasertib, AZD6738) and CHK1i (C, MK-8776) were purchased from Wuhan ChemNorm Biotech (Wuhan, China). The stock solutions of the inhibitors were prepared in 100% dimethyl sulfoxide (DMSO) and stored at − 80 °C for up to six months. RPMI 1640 culture medium, heat-inactivated fetal bovine serum (FBS), and trypsin–EDTA were obtained from Gibco (Thermo Fisher Scientific, Waltham, MA, USA). Chemicals and solvents were obtained from Sigma-Aldrich or Avantor Performance Materials Poland S.A. Other key reagents used in the studies are included in the “Materials and methods” section and Supplementary Table S1.

### Cell lines and treatment

Human HGSOC cell lines PEO1 with *BRCA2* truncating mutation (*BRCA2*^MUT^, c. 4965C>G, p.Y1655*) and PEO4 with *BRCA2* reversion mutation (*BRCA2*^REV^, 4965C>T, p.Y1655Y) were purchased from the European Collection of Authenticated Cell Cultures (ECACC, Salisbury, UK). A human HGSOC cell line with acquired resistance to olaparib and secondary *BRCA2* mutation PEO1-OR (*BRCA2*^MUT^, c.[4964A>T; 4965C>G], p.Y1655L) was developed by continuous exposure to incrementally increasing doses of olaparib as described previously^29^. Cells were cultured as monolayers in RPMI 1640 medium containing GlutaMAX™ supplement, HEPES, supplemented with 10% FBS and maintained in the cell culture incubator (37 °C, 5% CO_2_). Cells were subcultured using 0.1% trypsin solution with 0.4 mM EDTA.

Unless otherwise stated, during all experiments olaparib-sensitive cells (PEO1 and PEO4) were treated with the final concentration of 10 μM olaparib, 2.5 μM ATRi, and 1 μM CHK1i, whereas olaparib-resistant cells (PEO1-OR) were incubated with 15 μM olaparib, 7.5 μM ATRi and 2.5 μM CHK1i.

### MTT cell viability assay

Cell viability in response to tested inhibitors was evaluated with MTT assay. Cells were seeded in 96-well plates (1.0 × 10^4^ PEO1 or PEO1-OR cells and 2.0 × 10^4^ PEO4 cells per well for 2-day treatment, and 0.4 × 10^4^ PEO1 or PEO1-OR cells and 0.8 × 10^4^ PEO4 cells per well for five-day treatment) and incubated for 24 h (37 °C, 5% CO_2_). The next day culture medium was changed and 4 × concentrated working solutions of inhibitors or their combinations were added to wells for 2 or 5 days of incubation (37 °C, 5% CO_2_). Following the treatment, the medium was aspirated, 50 μL of MTT solution (0.5 mg/mL in DPBS) was added to each well, and the plates were incubated for 4 h (37 °C, 5% CO_2_). Afterward, 100 μL of DMSO was added to each well, plates were incubated at room temperature or 37 °C protected from light until complete solubilization of the formazan crystals, and the samples were mixed for about 30 s using a plate shaker.

The absorbance was determined spectrophotometrically on a microplate reader (Synergy HTX, BioTek, Shoreline, WA, USA) at an experimental wavelength of 580 nm, using 720 nm as a reference wavelength. To determine cell viability, the absorbance at 720 nm was subtracted from the absorbance at 580 nm (A_580_ − A_720_) for individual wells. Then, the mean absorbance value of background wells (DMSO with MTT) was subtracted from the absorbance readings of tested samples. Relative cell viability was calculated as the percentage of untreated control cells using corrected absorbance values. Each experiment was independently repeated four times (n = 4) with a minimum of three intraplate technical replicates and the results were presented as mean ± standard deviation (SD).

Coefficient of drug interaction (CDI) values were calculated using corrected absorbance values to determine the total cytotoxic effect of concurrent incubation with two inhibitors according to the formula:

$${\text{CDI}}=\frac{{\text{AB}}}{\mathrm{A }\times \mathrm{ B}}\times 100\mathrm{\%}$$

Based on the corrected absorbance of each group, AB is the cell viability of the two-agent combination group, and each A or B is the cell viability of the single-agent group. CDI values indicated whether the effect exerted by the combination of drugs was strongly synergistic (CDI < 0.30), synergistic (0.3 ≤ CDI < 0.7), moderately synergistic (0.7 ≤ CDI < 0.85), slightly synergistic (0.85 ≤ CDI < 1.0), additive (CDI = 1.00) or antagonistic (CDI > 1.00).

### Clonogenic assay

A clonogenic assay was performed to evaluate the ability of cells to proliferate and form colonies in the presence of studied inhibitors. Cells were seeded in 6-well plates (2 × 10^3^ cells/well) and allowed to attach for 24 h (37 °C, 5% CO_2_). The next day culture medium was changed and 100 × concentrated working solutions of inhibitors or their combinations were added to wells for 5 days of incubation (37 °C, 5% CO_2_). After the incubation period, the medium was refreshed (3 mL/well), and cells were incubated (37 °C, 5% CO_2_) in the absence of inhibitors for 7 days (PEO1-OR) or 10 days (PEO1, PEO4) until forming single, non-overlapping colonies. Finally, the colonies were fixed with the mixture of methanol and glacial acetic acid (7:1 (v/v)) for 10 min, stained with 1% (w/v) crystal violet in 20% (v/v) ethanol for 20 min, thoroughly rinsed with deionized water, and air-dried at room temperature. Stained colonies were photographed and counted manually. Colony formation efficiency (clonogenic survival) relative to control cells was calculated according to the formula:

$$\mathrm{Colony\; formation\; efficiency}=\frac{\mathrm{number \; of \; colonies \; in \; a \;treatment \;group}}{\mathrm{number \; of \; colonies \; in \;a \;control \;group}}\times 100\mathrm{\%}$$

Each experiment was independently repeated four times (n = 4) and the results were presented as mean ± SD. CDI values were calculated as mentioned above in “Materials and methods” (“MTT cell viability assay” section) based on clonogenic survival values for combination and single-agent groups.

### RNA isolation, cDNA synthesis, and quantitative real-time PCR

Gene expression profiling (*ATR*, *CASP3*, *CHEK1*, *PARP1*) was conducted using quantitative real-time PCR (qRT-PCR). Cells were seeded in 100 mm dishes (2 × 10^6^ cells) and incubated for 24 h (37 °C, 5% CO_2_). The next day culture medium was changed and 100 × concentrated working solutions of inhibitors or their combinations were added to each dish for 2 days (37 °C, 5% CO_2_). After the incubation period, cells were harvested by trypsinization, centrifuged (300×*g*, 5 min, 4 °C) and pellets were stored at − 80 °C. Total RNA was isolated and purified using mirVana™ miRNA Isolation Kit with phenol (Invitrogen™, Thermo Fisher Scientific) according to the manufacturer’s protocol and stored at − 80 °C. The quality and quantity of isolated RNA were analyzed by absorbance measurements at 230, 260, and 280 nm using a BioTek Eon™ microplate spectrophotometer.

For cDNA synthesis, 1000 ng of total RNA was reverse transcribed in 0.2 mL thin-walled tubes using High-Capacity cDNA Reverse Transcription Kit (Applied Biosystems™, Thermo Fisher Scientific) with RNase inhibitor and PTC-200 DNA Engine^®^ Cycler (MJ Research Inc., Canada) according to the manufacturer’s instruction and cDNA was stored undiluted at − 80 °C.

qRT-PCR was performed using 10 ng of obtained cDNA, TaqMan™ Universal Master Mix II with no UNG, and predesigned TaqMan™ Gene Expression Assays listed in Table S2 (Applied Biosystems™, Thermo Fisher Scientific) in the final volume of 10 μL and run in the Rotor-Gene Q 5plex HRM (QIAGEN Inc., Germany) according to the manufacturer's protocols. Raw data was analyzed using Q-Rex Software (QIAGEN Inc., Germany).

Three widely used potential endogenous control genes (*ACTB*, *GAPDH,* and *HPRT1*) were analyzed in all OC cell lines to assess the stability of their expression across different treatment conditions and to choose a reference gene for normalization in gene expression studies. RNA for each treatment condition from two independent experiments was reversely transcribed and obtained cDNA was subjected to qRT-PCR analysis in duplicate using the abovementioned experimental conditions. Averaged threshold cycle values were compared and used to rank the genes with RefFinder web-based tool^52^.

Relative mRNA expression was calculated according to the comparative 2^−∆∆Ct^ method using *ACTB* as a reference gene and presented as a fold-change relative to untreated control cells. Each experiment was independently repeated three to four times (n = 3–4) with each sample run in duplicate or triplicate (SD between technical replicates ≤ 0.3 cycles) and the results were presented as mean ± SD.

### Caspase 3/7 activity assay

The effect of studied inhibitors and their combinations on the activity of caspase 3 and caspase 7 (markers of apoptosis) was determined using the CellEvent™ Caspase-3/7 Green Flow Cytometry Assay Kit (Invitrogen™, Thermo Fisher Scientific) according to the manufacturer’s protocols. Cells were seeded in 100 mm dishes (2 × 10^6^ cells) and incubated for 24 h (37 °C, 5% CO_2_). The next day culture medium was changed and 100 × concentrated working solutions of inhibitors or their combinations were added to each dish for 2 days (37 °C, 5% CO_2_). Cells were also incubated with 2.5 μM camptothecin (CPT), which was used as a positive control for the induction of caspase 3 and 7 activity. After the incubation period, cells were harvested by trypsinization, centrifuged (300×*g*, 5 min, room temperature), resuspended in DBPS with 5% FBS, and incubated with CellEvent™ Caspase-3/7 Green Detection Reagent for 1 h at room temperature. SYTOX™ AADvanced™ Dead Cell Stain was added during the final 5 min of staining. Labeled cells were immediately acquired by collecting at least 10,000 events with BD™ LSR II flow cytometer (Becton Dickinson, San Jose, CA, USA).

Flow cytometric data were analyzed using FlowJo v.7.6.1 analyzing software (Ashland, OR, USA). Each experiment was independently repeated three to four times (n = 3–4) and the results were presented as mean ± SD.

### Apoptosis assay using annexin V-FITC/PI staining

Annexin V-FITC/propidium iodide (PI) staining was performed with Dead Cell Apoptosis Kits with Alexa Fluor™ 488 annexin V/PI for flow cytometry (Invitrogen™, Thermo Fisher Scientific) according to the manufacturer’s instruction to detect apoptosis in OC cells. Cells were seeded in 100 mm dishes (2 × 10^6^ cells) and incubated for 24 h (37 °C, 5% CO_2_). The next day culture medium was changed and 100 × concentrated working solutions of inhibitors or their combinations were added to each dish for 2 days (37 °C, 5% CO_2_). Cells were also incubated with 2.5 μM CPT, which was used as a positive control for the induction of apoptosis. After the incubation period, cells were harvested by trypsinization, centrifuged (300×*g*, 5 min, 4 °C), washed in ice-cold DPBS, resuspended in annexin-biding buffer containing Alexa Fluor™ 488 annexin V and PI provided with the kit, and incubated for 30 min at 4 °C. Labeled cells were immediately acquired by collecting at least 10,000 events with BD™ LSR II flow cytometer (Becton Dickinson, San Jose, CA, USA).

Flow cytometric data were analyzed using FlowJo v.7.6.1 analyzing software (Ashland, OR, USA). Each experiment was independently repeated three times (n = 3) and the results were presented as mean ± SD.

### DNA damage response antibody array

The expression of 27 DNA damage response-associated proteins was semi-quantitatively determined using a commercially available antibody array system RayBio^®^ C-Series Human DNA Damage Response Antibody Array 1 (RayBiotech Life, Inc., Peachtree Corners, USA) according to the manufacturer's instructions.

PEO1 and PEO1-OR cells were seeded in 100 mm dishes (2 × 10^6^ cells) and incubated for 24 h (37 °C, 5% CO_2_). The next day culture medium was changed and 100 × concentrated working solutions of inhibitors or their combinations were added to each dish for 2 days (37 °C, 5% CO_2_). After the incubation period, cells were washed twice with ice-cold DPBS and lysed directly on plates afterward using ice-cold cell lysis buffer with a protease inhibitor cocktail provided with the kit. Cell lysates were centrifuged (14,000×*g*, 5 min, 4 °C), supernatants were transferred to fresh microcentrifuge tubes, and clear lysates were stored at − 80 °C. The total protein concentration in each lysate was determined spectrophotometrically using Pierce™ BCA Protein Assay Kit (Thermo Fisher Scientific) and a Synergy HTX microplate reader (BioTek, Shoreline, WA, USA) according to the manufacturer's microplate procedure.

The antibody arrays were incubated with blocking buffer (30 min, room temperature) followed by the incubation with 1 mL of diluted samples containing 200 μg of total protein each (overnight, 4 °C). Thereafter, after repeated washings with the Wash Buffer I and II, membranes were subsequently incubated with a biotinylated antibody cocktail (overnight, 4 °C) and HRP-streptavidin (2 h, room temperature). Next, all membranes from each biological replicate were placed next to each other, and incubated with the detection buffer (2 min, room temperature), and the chemiluminescence signal was measured for 2 min with Azure 300 Imaging System (Azure Biosystems, Dublin, CA, USA) yielding non-overlapping signals.

Densitometric data were analyzed according to the manufacturer's instructions. Briefly, the integrated density of each antigen-specific antibody spot was measured using the same circle (area and shape) on every array with ImageJ software (NIH, Bethesda, MD, USA). After correcting the raw numerical densitometry data by subtracting background (negative control spots), densitometric data were further normalized to the positive control signals from reference arrays with control cells. The changes in protein expression were calculated as a fold-change relative to untreated control cells. Each experiment was independently repeated two times (n = 4) with two technical replicates on each membrane and the results were presented as mean ± SD.

### Statistical analysis

Statistical analysis was performed with GraphPad Prism version 9.5.1 for Windows (GraphPad Software, San Diego, CA, USA) using the tests specified in the Results section for each experiment. Shapiro–Wilk or D’Agostino–Pearson tests were used to assess whether data come from a normal distribution. Homogeneity of variance within groups was evaluated with Brown–Forsythe or Bartlett’s tests. Ordinary one-way ANOVA followed by Tukey’s multiple comparison tests was used to assess the statistical significance of differences where three independent groups were defined by one factor (cell line). Two-way ANOVA followed by Tukey’s or Šídák multiple comparison tests was used to assess the statistical significance of differences where three independent groups were defined by two factors (drug treatment and cell line or drug treatment and time of incubation). Differences among groups were considered statistically significant at: ^^^*p* < 0.05, ^^^^*p* < 0.01, ^^^^^*p* < 0.001, ^^^^^^*p* < 0.0001 (comparison between cell lines); *p < 0.05, **p < 0.01, ***p < 0.001, ****p < 0.0001 (treatment vs. control); ^+^*p* < 0.05, ^++^*p* < 0.01, ^+++^*p* < 0.001, ^++++^*p* < 0.0001 (olaparib vs. combination with ATRi or CHK1i); ^#^*p* < 0.05, ^##^*p* < 0.01, ^###^*p* < 0.001, ^####^*p* < 0.0001 (ATRi or CHK1i vs. respective combinations with olaparib).

### Supplementary Information

Supplementary Information.

## Data Availability

The datasets generated during and/or analysed during the current study are available from the corresponding author on reasonable request.

## Abbreviations

A: ATR inhibitor (ceralasertib)
ATR: Ataxia telangiectasia and RAD3-related protein
ATRi: ATR inhibitor(s)
BER: Base excision repair
BRCA1: Breast cancer type 1 susceptibility protein
BRCA2: Breast cancer type 2 susceptibility protein
C: CHK1 inhibitor (MK-8776)
CDI: Coefficient of drug interaction
CHK1: Checkpoint kinase 1
CHK1i: CHK1 inhibitor(s)
CHK2: Checkpoint kinase 2
CPT: Camptothecin
DDR: DNA damage response
DR: Direct repair
DSB: Double-strand break
ECACC: European Collection of Authenticated Cell Cultures
ERCC1: Excision repair cross-complementation group 1
FBS: Fetal bovine serum
HGSOC: High-grade serous ovarian cancer
HR: Homologous recombination
MDM2: Mouse double minute 2
MGMT: O^6^-methylguanine-DNA methyltransferase
MMR: Mismatch repair
NBS1: Nijmegen breakage syndrome protein 1
NER: Nucleotide excision repair
NHEJ: Non-homologous end joining
O: Olaparib
OC: Ovarian cancer
OPTN: Optineurin
PARP1: Poly(ADP-ribose) polymerase 1
PARPi: PARP inhibitor(s)
PEO1-OR: PEO1 olaparib-resistant cell line
PLK1: Polo-like kinase 1
SD: Standard deviation
SSB: Single-strand break
