# Supplementary material for: Targeted inhibition of the ATR/CHK1 pathway overcomes resistance to olaparib and dysregulates DNA damage response protein expression in BRCA2MUT ovarian cancer cells
Source: Sci Rep. 2023 Dec 19;13:22659. doi: 10.1038/s41598-023-50151-y (PMC10730696; doi:10.1038/s41598-023-50151-y)
Supplement: Supplementary file 1 — Supplementary Information. [file 41598_2023_50151_MOESM1_ESM.pdf]

Supplementary Information

Targeted inhibition of the ATR/CHK1 pathway overcomes resistance to olaparib and dysregulates DNA damage response protein expression in *BRCA2*^MUT^ ovarian cancer cells

Łukasz Biegała[1,2], Arkadiusz Gajek[1], Izabela-Szymczak-Pajor[3], Agnieszka Marczak[1], Agnieszka Śliwińska[3], Aneta Rogalska[1,*]

Supplementary Figure 1 (Figure S1)

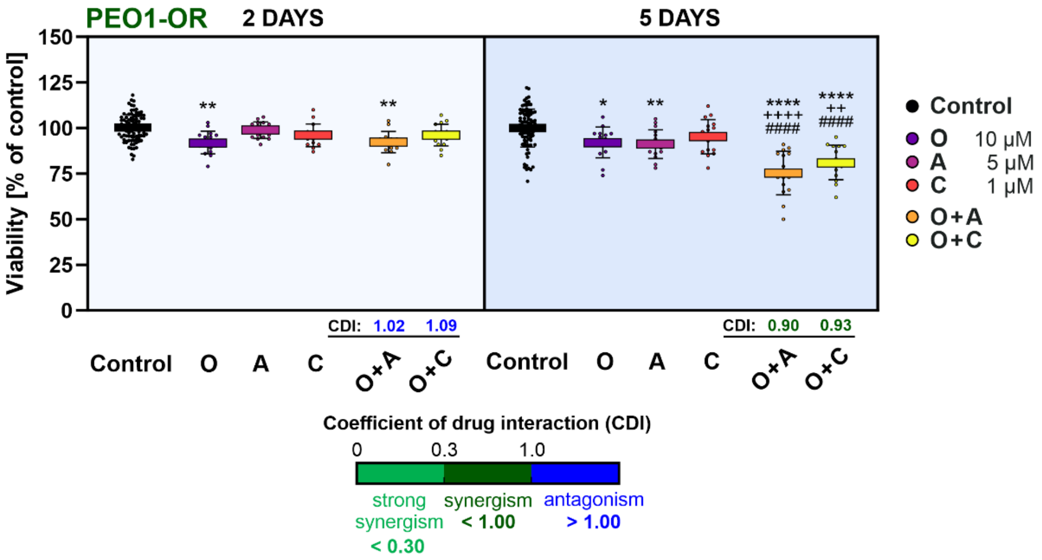

Figure S1. Viability of PEO1-OR cells after incubation with studied inhibitors (O, A, C) or their combinations (O + A, O + C) at designated concentrations optimal for olaparib-sensitive cells (PEO1 and PEO4) for 2 days and 5 days assessed by MTT assay. Coefficient of drug interaction (CDI) values were calculated to evaluate the interaction effect of combination treatments. Data were expressed as mean ± SD (n = 4). Statistical significance was assessed using two-way ANOVA followed by Tukey's multiple comparison test: $^{*}p < 0.05$, $^{**}p < 0.01$, $^{****}p < 0.0001$ (treatment vs. control); $^{++}p < 0.01$, $^{++++}p < 0.0001$ (olaparib vs. combination with ATRi or CHK1i); $^{\#\#\#\#}p < 0.0001$ (ATRi or CHK1i vs. respective combinations with olaparib).

Supplementary Figure 2 (Figure S2)

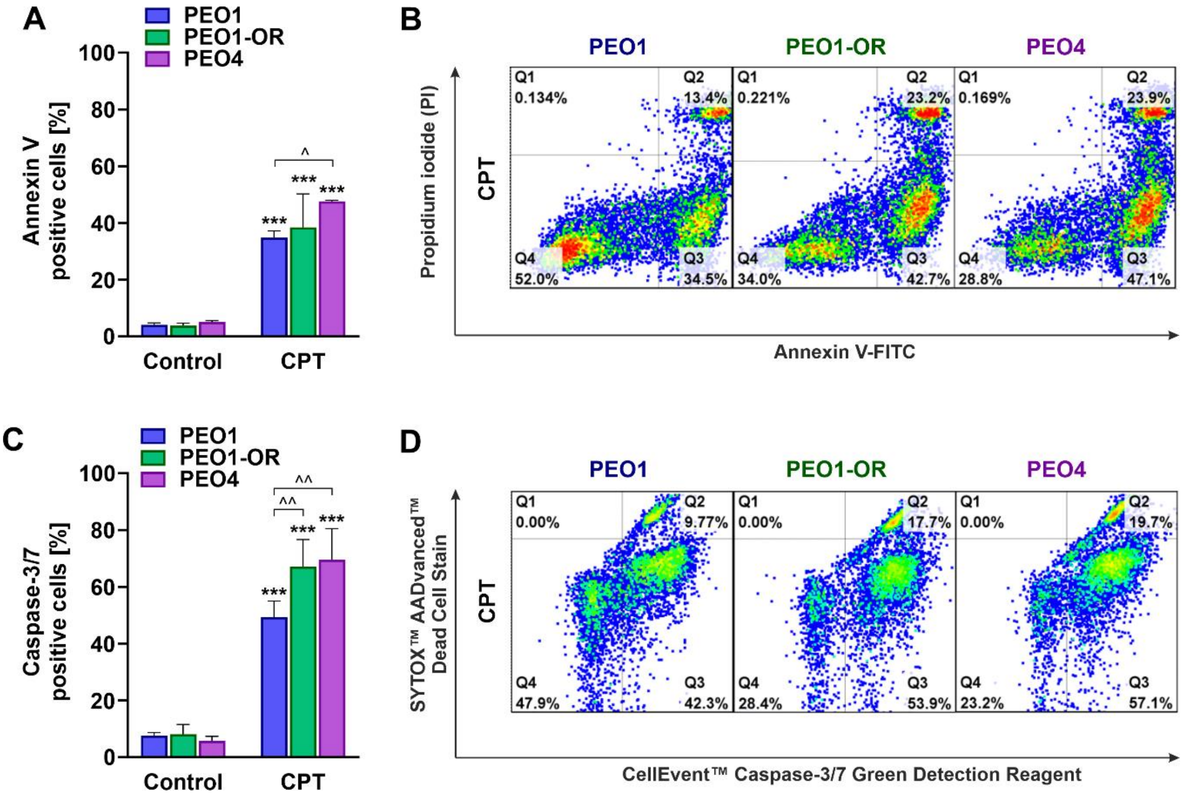

Figure S2.
(A) Apoptosis in OC cells was examined by dual staining with annexin V-FITC and PI in response to incubation with 2.5 μM camptothecin (CPT) for 2 days as a positive control. Apoptosis rate was presented as a percentage of annexin V positive (apoptotic) cells relative to untreated control cells. Data are presented as mean ± SD (n = 3).
(B) Representative dot plots of annexin V-FITC and PI-stained OC cells with the indicated percentage of non-viable cells (Q1), necrotic cells (Q2), apoptotic cells (Q3), and viable cells (Q4).
(C) Activation of caspase 3 and caspase 7 in apoptotic OC cells examined by dual staining using CellEvent™ Caspase-3/7 Green Flow Cytometry Assay Kit in response to incubation with 2.5 μM CPT for 2 days as a positive control. Caspase-3/7 activity was presented as a percentage of caspase-3/7 positive cells relative to untreated control cells. Data are presented as mean ± SD (n = 3).
(D) Representative dot plots of a two-parameter apoptosis assay for detection of activated caspase-3/7 (CellEvent™ Caspase-3/7 Green Detection Reagent) and the distinction between live and dead cell (SYTOX™ AADvanced™ Dead Cell Stain) with the indicated percentage of necrotic cells (Q2), viable cells (Q3), apoptotic cells with activated caspase-3/7 (Q4). Statistical significance was assessed using two-way ANOVA followed by Šídák multiple comparison test: $^{\wedge}p < 0.05$, $^{\wedge\wedge}p < 0.01$ (comparison between cell lines); $^{***}p < 0.001$ (treatment vs. control).

Supplementary Figure 3 (Figure S3)

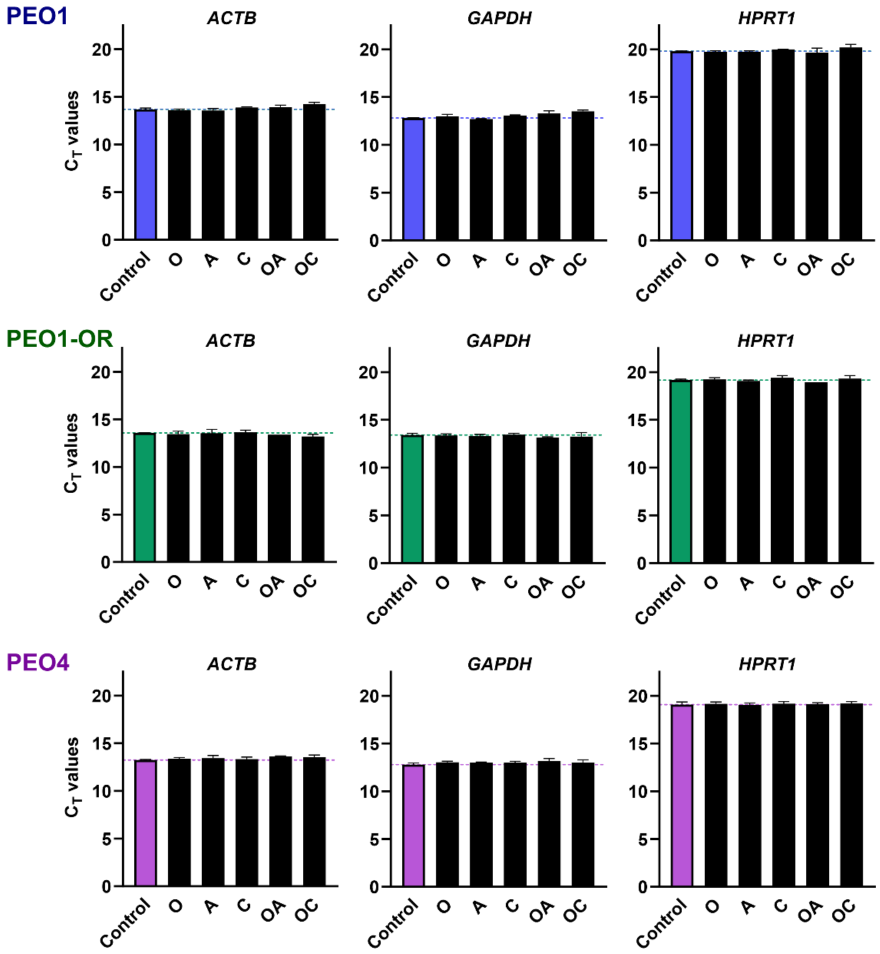

Figure S3. Evaluation of three candidate reference genes (*ACTB*, *GAPDH*, *HPRT1*) in OC cell line for the normalization of RT-qPCR gene expression. Graphs represent variations in threshold cycle values (C_T) of tested genes across different treatment conditions as mean ± SD (n = 2). Dashed lines indicate mean C_T values for untreated control cells. The expression stability of candidate genes was evaluated based on differences in C_T values between treatments and RefFinder web-based tool.

Supplementary Figure 4 (Figure S4)

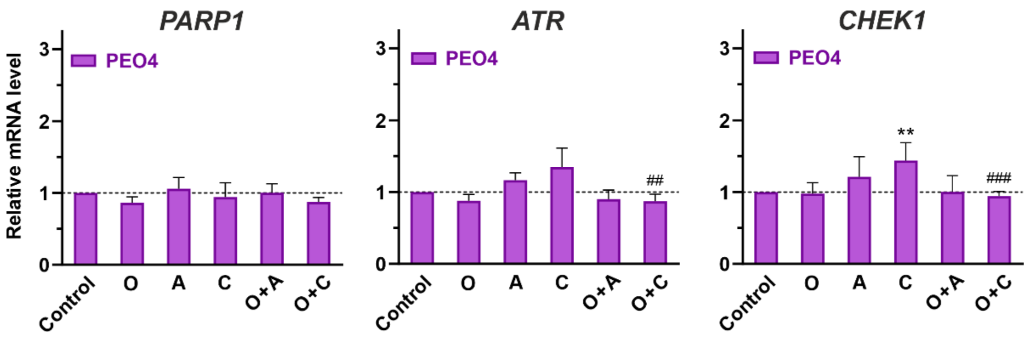

Figure S4. Quantitative analysis of the mRNA expression of *PARP1*, *ATR*, and *CHEK1* in PEO4 cells. OC cells were treated with studied inhibitors (O, A, C) or their combinations (O + A, O + C) for 2 days and mRNA levels were determined by real-time qPCR. Data were normalized to β-actin and expressed relative to untreated cells as mean ± SD (n = 4). Statistical significance was assessed using two-way ANOVA followed by Tukey's multiple comparison test: $^{**}p < 0.01$ (treatment vs. control); $^{\#\#\#}p < 0.001$ (ATRi or CHK1i vs. respective combinations with olaparib).

Supplementary Table 1 (Table S1)

List of key reagents used in the study.

Reagent	Catalog Number	Manufacturer
(S)-(+)-Camptothecin	C9911	Sigma-Aldrich
CellEvent™ Caspase-3/7 Green Flow Cytometry Assay Kit	C10427	Invitrogen™ (Thermo Fisher Scientific)
Ceralasertib	TBW02661	Wuhan ChemNorm Biotech
Crystal violet	911517ZA	VWR
FBS, heat-inactivated, qualified	10270106	Gibco (Thermo Fisher Scientific)
High-Capacity cDNA Reverse Transcription Kit	4368814	Applied Biosystems™ (Thermo Fisher Scientific)
mirVana™ miRNA Isolation Kit, with phenol	AM1560	Invitrogen™ (Thermo Fisher Scientific)
MK-8776	TBW02666	Wuhan ChemNorm Biotech
MTT	20395.03	SERVA Electrophoresis
Olaparib	S1060	Selleck Chemicals
Pierce™ BCA Protein Assay Kit	23225	Thermo Fisher Scientific
RayBio® C-Series Human DNA Damage Response Antibody Array 1	AAH-DDR-1-8	RayBiotech
RNase Inhibitor	N8080119	Applied Biosystems™ (Thermo Fisher Scientific)
RPMI 1640, GlutaMAX™ Supplement, HEPES	72400-021	Gibco (Thermo Fisher Scientific)
TaqMan™ Gene Expression Assay (FAM)	4331182	Applied Biosystems™ (Thermo Fisher Scientific)
TaqMan™ Universal Master Mix II, no UNG	4440049	Applied Biosystems™ (Thermo Fisher Scientific)
Trypsin-EDTA	25200072	Gibco (Thermo Fisher Scientific)

Supplementary Table 2 (Table S2)

List of TaqMan™ Gene Expression Assay used for gene expression studies (RT-qPCR).

Gene	Assay ID
ACTB	Hs01060665_g1
ATR	Hs00992123_m1
CASP3	Hs00234387_m1
CHEK1	Hs00967506_m1
PARP1	Hs00242302_m1
HPRT1	Hs02800695_m1
GAPDH	Hs02758991_g1
